# Improved effectiveness of performance monitoring in amateur instrumental musicians^☆^

**DOI:** 10.1016/j.neuropsychologia.2013.09.025

**Published:** 2014-01

**Authors:** Ines Jentzsch, Anahit Mkrtchian, Nayantara Kansal

**Affiliations:** School of Psychology & Neuroscience, University of St Andrews, St Andrews KY16 9JP, UK

**Keywords:** Amateur musicians, Instrumental practice, Executive functions, Response monitoring, Conflict, Error-related negativity

## Abstract

Here we report a cross-sectional study investigating the influence of instrumental music practice on the ability to monitor for and respond to processing conflicts and performance errors. Behavioural and electrophysiological indicators of response monitoring in amateur musicians with various skill levels were collected using simple conflict tasks. The results show that instrumental musicians are better able than non-musicians to detect conflicts and errors as indicated by systematic increases in the amplitude of the error-related negativity and the N200 with increasing levels of instrumental practice. Also, high levels of musical training were associated with more efficient and less reactive responses after experience of conflicts and errors as indicated by reduced post-error interference and post-conflict processing adjustments. Together, the present findings suggest that playing a musical instrument might improve the ability to monitor our behavior and adjust our responses effectively when needed. As these processes are amongst the first to be affected by cognitive aging, our evidence could promote musical activity as a realistic intervention to slow or even prevent age-related decline in frontal cortex mediated executive functioning.

## Highlights

• We evaluated the effects of instrumental practice on cognitive control functions.
• We show that Instrumental practice affects the ability to detect errors and conflicts.
• We found an enlarged ERN and N200 in musicians compared to non-musicians.
• Musical training predicted less reactive control adjustments after conflicts/errors.

## 1 Introduction

Music plays an important role in virtually all societies. Nevertheless, in times of economic hardship, funds for music education are often amongst the first to be cut (e.g., Shepherd, 2011). This is particularly worrying given both anecdotal and limited research evidence suggesting that music can have strong positive effects on our physical as well as psychological functioning. In the present study, we want to specifically focus on the benefits that active engagement with music, even at an amateur level, might exert on executive control. Executive functions associated with frontal brain areas such as action planning, goal-orientation, the inhibition of inappropriate responses, and response monitoring, show greater and earlier decline with age than other brain functions (for reviews, see Jurado and Rosselli, 2007, Raz et al., 2005, West, 1996). It has recently been suggested that highly skilled motor performance such as that achieved through intensive musical practice might have the capacity to enhance executive functions in both, children and adults, and might delay cognitive aging (e.g. Bialystok and DePape, 2009, Hanna-Pladdy and MacKay, 2011; but see Schellenberg, 2011). In addition, Sluming et al. (2002) reported age-related volume reduction in frontal cortex areas to be smaller in musicians than non-musicians. These findings are particularly interesting as they might indicate that age-related decline in executive frontal cortex functioning can potentially be delayed or even prevented by continuous engagement in complex motor activity as is required for instrumental practice in musicians (e.g., Sluming et al., 2002).

Both professional as well as advanced amateur musicians spend a substantial amount of time on instrumental practice, with the amount of practice time being one of the best predictors for musical skill achievement (Ericsson et al., 1993, Jaencke, 2009, Levitin, 2006, Krampe and Ericsson, 1996, Sloboda et al., 1996). For example, concert-level performers have an accumulated practice time of over 10,000 h within a 10-year period (e.g., Gruhn, Galley, & Kluth, 2003).

It is not surprising that such extensive motor skill practice does result in substantial structural as well as functional brain adaptations. Both cross-sectional as well as longitudinal studies have shown increased primary motor area representations, depending on the specific type of skill practice (e.g., Amunts et al., 1997, Pascual-Leone, 2001; for a review, see Herholz & Zatorre, 2012). These anatomical changes are assumed to enable musicians to control their movements more efficiently (e.g., Muente, Altenmueller, & Jaencke, 2002). More importantly for present purposes, frontal cortex functions also seem to be strongly altered in professional musicians compared to non-musicians (e.g., Muente, Nager, Beiss, Schroeder, & Altenmueller, 2003). Music performance involves a number of executive functions such as selective attention and inhibitory control (e.g. Bialystok & Depape, 2009) that require the involvement of frontal brain structures. For example, during performance, musicians need to continuously monitor their executed responses using both auditory and proprioceptive feedback, in order to rapidly adjust subsequent movements in the event of undesired response outcomes (e.g., Schlaug, Norton, Overy, & Winner, 2005; for a review see also Zatorre, Chen, & Penhune, 2007).

An influential theory about how frontal cortex mediated response monitoring is exercised is the conflict monitoring theory, recently put forward by Botvinick, Braver, Barch, Carter, and Cohen (2001). The authors postulate the existence of a system within the anterior cingulate cortex (ACC) that continuously evaluates the amount of conflict in information processing. The conflict monitoring system transfers this information to the centers of the brain that are responsible for adjustments of cognitive control. Control is increased when conflict is detected, by enhancing the level of attention towards task-relevant aspects of the stimulus environment, therefore reducing further conflict. Interference tasks such as the Eriksen and Eriksen (1974) task, the Simon (1990) task or the Stroop (1935) task are specifically informative, as they allow the study of both evaluative control (detection of conflicts and errors) and executive control (implementation of changes in behavior or brain activity after the experience of conflict or errors). In these experimental paradigms a task-relevant and a task-irrelevant stimulus feature can potentially activate competing responses, resulting in conflict between simultaneously activated response alternatives. Several authors have used these interference tasks to study effects of instrumental practice on executive functions, and generally report improved inhibitory control in participants engaging in instrumental practice. For example, Bialystok and DePape (2009) reported a reduced interference effect (reaction time difference between incongruent and congruent Stroop trials) in instrumental musicians compared to non-musicians in an auditory Stroop task that required participants to judge the relative pitch of two spatial word descriptors (“high” and “low”). The authors also reported musicians to be generally faster than non-musicians in the Simon task, but no difference in the size of the congruency effect between groups was observed in this task. Travis, Harung, and Lagrosen (2011) replicated Bialystok and DePape's (2009) results of a reduced Stroop interference effect in professional musicians compared to non-musicians, using a visual color-word Stroop task. Furthermore, longitudinal studies suggest that there is indeed a causal link between musical training and improved inhibitory control (Dege et al., 2011, Moreno et al., 2011).

Although the studies mentioned above have suggested an enhanced ability in musicians to inhibit task-irrelevant information, it remains unclear how cognitive control functions are generally altered in musicians. Furthermore, it remains unclear how these behavioral findings relate to underlying changes in brain response. Limited evidence suggests that instrumental musicians might have better response monitoring skills, as indicated by faster error detection and correction speed in intermediate and advanced piano players (e.g., Palmer & Drake, 1997). Furthermore, researchers have suggested that expert pianists can detect errors before the execution of the incorrect response, as indicated by the presence of pre-error negativity and delayed as well as less forceful error responses (e.g., Ruiz et al., 2009, Maidhof et al., 2009). However, other studies indicate that the ability to detect errors in advance of the response execution might not be specific to highly skilled musicians. For example, Gehring, Goss, Coles, Meyer, and Donchin (1993) reported, in a general population sample, a negative correlation between error response force and the amplitude of the error-related negativity (ERN; Falkenstein et al., 1991, Gehring et al., 1993), an electrophysiological brain correlate associated with the detection of incorrect responses and assumed to originate from anterior cingulate cortex (ACC). This finding indicates that on trials with active error monitoring (large ERN) the error response could already be partially inhibited by the time it was executed (reduced error force).

The present study therefore aims to evaluate differences in cognitive control functions between non-musicians and amateur musicians, more specifically, the ability to monitor behavior and to implement necessary control adjustments. Implementation of control adjustments are quantified here as (A) the size of the Gratton effect, that is, the reduction in interference effects after the experience of conflict in the previous trial (e.g., Gratton et al., 1992, Kerns et al., 2004, Stuermer et al., 2002); (B) as the amount of response slowing after errors (e.g. Laming, 1968, Jentzsch and Dudschig, 2009). We used two established cognitive interference tasks, the Simon and the Stroop task. These paradigms are well suited to study both participants’ ability to detect interfering information, and the effectiveness of remedial actions after interference detection (e.g., Kerns et al., 2004). Furthermore, we collected neurophysiological data of frontal brain functioning in order to link behavioural effects with underlying changes in brain function. More specifically, two components of the event-related brain potentials were analyzed: the amplitude of the N200 component (e.g. Yeung, Botvinick, & Cohen, 2004), an electrophysiological marker of conflict strength, and the amplitude of the ERN as an index of error detection.

We predict that intensive musical practice will not only be associated with an increased ability to detect processing conflicts and errors, but also an improved effectiveness of control adjustments after error and conflict experience.

## 2 Method

### 2.1 Participants

Thirty-six young adults were divided into four groups (8–10 participants per group) according to the number of accumulated hours of practicing a musical instrument over their lifetime: ‘High’ (>5000 h), ‘Intermediate’ (2000 to 5000 h), ‘Low’ (200 to 2000 h) and ‘No’ (<200 h), see also Table 1 and Appendix. They were tested in a single session each, lasting approximately two hours. All participants had normal or corrected-to-normal vision, gave written informed consent, and received reimbursement of £10.

**Table 1:** Participant characteristics.

	Musical practice level				Statistics
	High	Intermediate	Low	No	*F*-value
Sex (female; male)	7; 1	6; 3	7; 2	8; 2	
Handedness (left; right)	1; 7	3; 6	0; 9	2; 8	
Age [years]	22.6	23.3	22.2	23.6	0.12
Years of education [years]	17.4	17.4	17.1	16.7	0.07
Accumulated practice time [h]	6275	2990	994	3	105.67⁎⁎⁎
Total years played [years]	14.5	9.3	8.1	0.1	27.01⁎⁎⁎
Musical knowledge score	17.9	15.7	12.4	7.4	19.53⁎⁎⁎
Physical activity [h/month]	5.6	15.1	15.8	21.4	1.67
PANAS score (positive)	33.5	34.1	31.3	33.2	0.36
PANAS score (negative)	17.1	16.6	18.9	17.0	0.18


Main musical instrument played	
Musical practice level	
High	5 Piano, 1 Organ, 2 Flute
Intermediate	5 Piano, 1 Harp, 1 Trumpet, 1 Flute, 1 Violin
Low	4 Piano, 1 Flute, 1 Saxophone, 1 Guitar, 1 Recorder, 1 Viola
No	n.a.

⁎⁎⁎ *p*<0.001.

### 2.2 Stimuli and apparatus

Participants completed the trait version of the PANAS Mood Questionnaire (Watson, Clark, & Tellegen, 1988) and a demographic music questionnaire (see Appendix) that also included questions relating to their musical skill level (lifetime accumulated music practice hours, total years played, start age, exams/grades achieved), and about general musical knowledge. General musical knowledge (maximal score 20) was assessed on a five-point scale using four items (ability to read music, knowledge of music history, knowledge of music theory, overall musical ability).

In the Stroop task, participants were presented with the words “blue” and “red” printed in either blue or red ink color and were asked to respond to the ink color (blue or red). In the Simon task, one of two stimulus shapes (square or diamond) was presented in black either to the left or the right (0.8 degrees visual angle) of the fixation point, and participants were asked to respond to the stimulus identity. Assignment of stimulus color (red or blue; Stroop task) or stimulus identity (diamond or square; Simon task) to response side was counterbalanced across participants. Compatible (C) and incompatible (IC) trials were presented with equal probability. The stimulus size (shapes and letters) was approximately 7×7 mm. Stimuli were presented on a 17-inch CRT monitor at a viewing distance of about 80 cm. Responses were recorded with two response keys mounted 15 cm apart in horizontal direction. Participants used the index finger of each hand to indicate their response on an ERTS keypad.

EEG activity was recorded using a BIOSEMI Active-Two amplifier with 72 Ag/AgCl electrodes. Four of these electrodes were placed at the outer canthi of and below each eye to record electro-occulographic activity. The usual ground electrode was replaced by a Common Mode Sense (CMS) active electrode and driven right leg (DRL) passive electrode feedback loop. The CMS also served as the recording reference. Data were recorded at a sampling rate of 256 Hz.

### 2.3 Procedure and design

Participants performed both the Stroop and the Simon task, with task order balanced across participants. For each of the two tasks, participants completed one practice block (10 trials) followed by six blocks (3 practice trials and 96 experimental trials in each block). Blocks were separated by short breaks where feedback about mean reaction time (RT) and error rate was provided. Stimuli were presented until response or for a maximum of 2000 ms, followed by a 1200 ms blank interval before the start of the next trials. Stimuli were presented randomly and with equal probability. At the end of the experiment, all participants completed both the PANAS and the demographic music questionnaire.

### 2.4 Data analysis

Trials with RTs smaller than 100 ms and larger than 1500 ms in trials *N*-1 and/or trial *N* were removed from the analysis (1.8%). Post-error choice error rates were determined by dividing the number of errors following an error by the total number of posterror trials, multiplied by one hundred. Post-correct choice error rates were determined by dividing the number of errors following correct responses by the total number of postcorrect trials, multiplied by one hundred.

To investigate group differences in behavioral conflict adjustments, a mixed ANOVA including between-subjects factor musical practice level (no, low, intermediate, high) and within-subjects factors current compatibility (compatible, incompatible), previous compatibility (compatible, incompatible) and task (Stroop, Simon) was conducted on mean RTs and arcsine-transformed (post-correct) choice error rates. For RT analysis only correct trials preceded by a correct trial were considered.

To investigate group differences in posterror behavior, a mixed ANOVA including between-subjects factor musical practice level (no, low, intermediate, high) and within-subjects factors trial type (post-error vs. post-correct) and task (Stroop, Simon) was conducted on mean RTs and arcsine-transformed choice error rates. Three participants had to be excluded from this analysis (one each from the high, low and no practice group) as they had too few trials per analysis cell (*N*<5).

The continuous EEG recording was segmented into stimulus- and response-locked epochs. Stimulus-locked epochs had a total duration of 1200 ms, starting 200 ms before stimulus onset. Response-locked epochs had a total duration of 1800 ms, starting 1000 ms before the keypress response. Trials containing amplitudes larger than 100 μV, a gradient larger than 75 μV and trials with a signal lower than 0.032 μV were removed from analysis (19%). Blinks and horizontal eye movements were corrected using the adaptive artifact correction method of Brain Electromagnetic Source Analysis (BESA, Version 5.0.6) software. Epochs were re-referenced off-line to average reference. A 0.1 Hz low cutoff and a 20 Hz high cutoff filter were applied. One participant (from the high level practice group) had to be excluded from EEG analyses due to a recording fault.

The mean amplitude of the N200 component in a 50 ms time window around the peak latency of the grand mean (260–310 ms) was analyzed in difference waves (incompatible minus compatible trials) at electrodes FCz and Cz in stimulus-locked data relative to a 100 ms baseline starting 100 ms before stimulus onset. A mixed ANOVA including between-subjects factor musical practice level and within-subjects factors electrode (FCz, Cz) and task (Simon, Stroop) was used to analyse N200 amplitudes. Only trials with correct responses were included in this analysis.

ERN peak amplitudes were measured in difference waves (errors minus correct response) at electrodes FCz and Cz in response-locked data, using a 100 ms baseline starting 150 ms before response onset, with automatic peak detection carried out in a search window 0 to 100 ms relative to response onset. For this analysis we averaged across the two tasks in order to achieve sufficiently high trial numbers. For ERN analysis the mean number of artifact-free trials per analysis cell was *N*=62 (minimum *N*=5, with *N*<10 for only two participants). A mixed ANOVA including between-subjects factor musical practice level and within-subjects factor electrode (FCz, Cz) was conducted on ERN peak amplitudes.

Group effects in the different dependent measures were assessed by testing for a linear trend across the four levels of group using the polynomial contrasts function in SPSS, as our a priori prediction was that observed effects should systematically relate to the amount of musical practice.

## 3 Results

### 3.1 Behavioural data

#### 3.1.1 Interference effects

##### 3.1.1.1 RTs

**Fig. 1:**
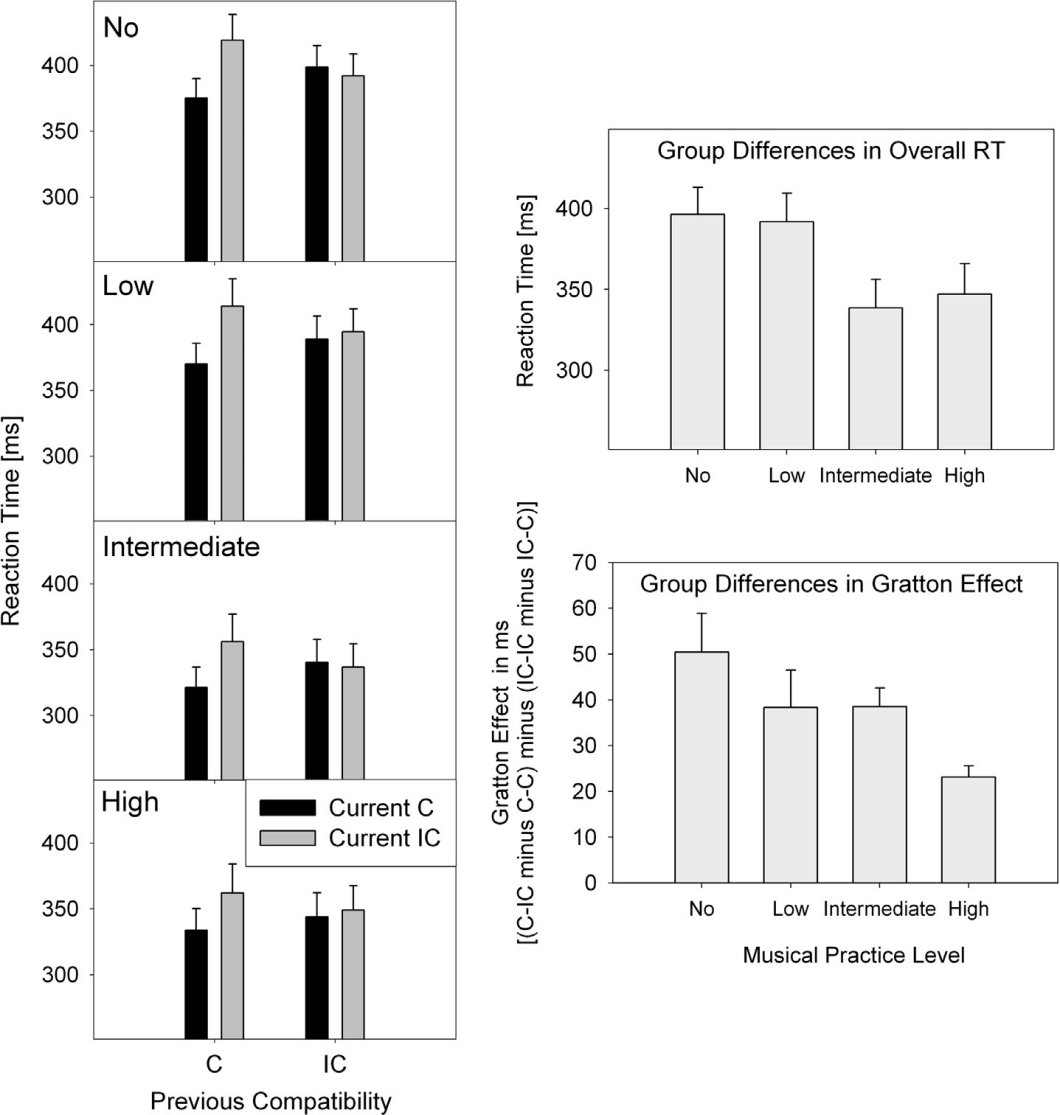
Left panel: mean RTs with previous and current compatibility superimposed in each graph, and musical practice level (no, low, intermediate, high) in separate graphs (top to bottom). The top right panel shows the averaged mean RT and the bottom right panel the average Gratton effect as a function of musical practice level.

RTs decreased with increasing levels of instrumental music practice (M[no, low, intermediate, high]=396, 392, 339, and 347 ms), *F*(linear: 1,32)=6.39, *p*=.017. There was a main effect of task, *F*(1,32)=27.96, *p*<.001, *η*_*p*_^2^=.47, due to overall faster RTs in the Stroop task (352 ms) than the Simon Task (385 ms). Participants were faster on compatible (359 ms) than incompatible trials (378 ms), *F*(1,32)=84.42, *p*<.001, *η*_*p*_^2^=.73. Task interacted with previous compatibility, *F*(1,32)=5.36, *p*=.027, *η*_*p*_^2^=.14. Task also interacted with current compatibility, *F*(1,32)=5.91, *p*=.021, *η*_*p*_^2^=.16, due to the compatibility effect (IC minus C) being larger in the Simon task (25 ms) than in the Stroop task (13 ms). Indicating the presence of the Gratton effect, there was a significant interaction between previous and current compatibility, *F*(1,32)=113.49, *p*<.001, *η*_*p*_^2^=.78, due to larger compatibility effects when the previous trial was compatible (38 ms) than when the previous trial was incompatible (0 ms). Importantly, this effect was modulated by musical practice level^1^, indicating a decrease in the Gratton effect with increasing levels of musical skills, *F*(linear: 1,32)=6.65, *p*=.015, see also Fig. 1. Finally, there was a three-way interaction between task, previous and current compatibility, *F*(1,32)=86.87, *p*<.001, *η*_*p*_^2^=.73. The Gratton effect ([C–IC minus C–C] minus [IC–IC minus IC–C]) was larger in the Simon task (50 ms) than in the Stroop task (11 ms).

##### 3.1.1.2 Choice error rates

Participants made fewer errors in the Stroop task (5.1%) than the Simon task (7.9%), *F*(1,32)=12.71, *p*=.001, *η*_*p*_^2^=.28. There was a main effect of previous compatibility, *F*(1,32)=36.15, *p*<.001, *η*_*p*_^2^=.53. There was also a main effect of current compatibility, *F*(1,32)=56.55, *p*<.001, *η*_*p*_^2^=.64, due to participants making fewer errors in compatible (4.9%) than incompatible trials (8.1%). There was an interaction between task and previous compatibility, *F*(1,32)=4.23, *p*=.048, *η*_*p*_^2^=.12, as well as an interaction between task and current compatibility, *F*(1,32)=13.37, *p*=.001, *η*_*p*_^2^=.30, due to a larger compatibility effect (IC minus C) in the Simon task (5.5%) than in the Stroop task (1.0%). There was a significant interaction between previous and current compatibility, *F*(1,32)=202.70, *p*<.001, *η*_*p*_^2^=.86, due to larger compatibility effects when the previous trial was compatible (8.0%) compared to when the previous trial was incompatible (−1.5%), indicating the presence of a Gratton effect. Importantly, this effect was modulated by musical practice level, indicating a decrease in the Gratton effect with increasing levels of musical skills, *F*(linear: 1,32)=6.70, *p*=.014. Finally, there was a three-way interaction between task, previous and current compatibility, *F*(1,32)=42.80, *p*<.001, *η*_*p*_^2^=.57, that is, the Gratton effect ([C–IC minus C–C] minus [IC–IC minus IC–C]) was larger in the Simon task (15.7%) than in the Stroop task (3.2%).

#### 3.1.2 Post-error effects

##### 3.1.2.1 RTs

Post-error trials were significantly slower (414 ms) than post-correct trials (358 ms), *F*(1,29)=71.06, *p*<.0001, *η*_*p*_^2^=.71. There was an interaction between trial type and task, *F*(1,29)=22.09, *p*<.001, *η*_*p*_^2^=.43, due to the post-error slowing effect (posterror minus postcorrect) being larger in the Stroop task (78 ms) than the Simon task (32 ms). Importantly this effect was further modulated by musical practice level, *F*(linear: 1,29)=5.42, *p*=.027; post-error slowing decreased with increasing levels of musical skill in the Stroop task, *F*(linear: 1,29)=5.65, *p*=.024, but not in the Simon task, *F*=0.49, n.s.

##### 3.1.2.2 Choice error rates

**Fig. 2:**
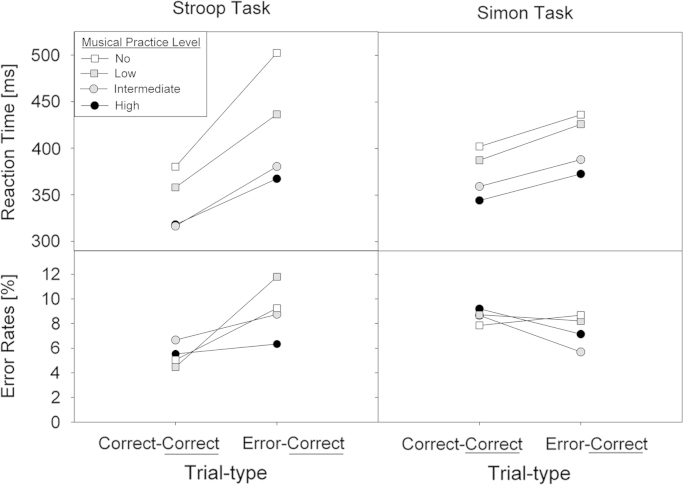
Mean RTs (top panel) and choice error rates (bottom panel) as a function of Trialtype (post-correct vs. post-error) and musical practice level. The data for the Stroop task are depicted in the left panel and the data for the Simon task in the right panel.

There was a significant interaction between trial type and task, *F*(1,29)=10.64, *p*=.003, *η*_*p*_^2^=.27. Participants made more errors on post-error (9.0%) than post-correct trials (5.4%) in the Stroop task (*p*=.036), but less errors on post-error (7.4%) than post-correct trials (8.6%) in the Simon task (*p*=.027). Important for present purpose, the effect of trialtype was modulated by musical practice level, *F*(linear: 1,29)=4.80, *p*=.037, indicating increasingly less compromised posterror accuracy with increasing levels of musical skill (see also, Fig. 2).

### 3.2 Electrophysiological data

#### 3.2.1 N200

**Fig. 3:**
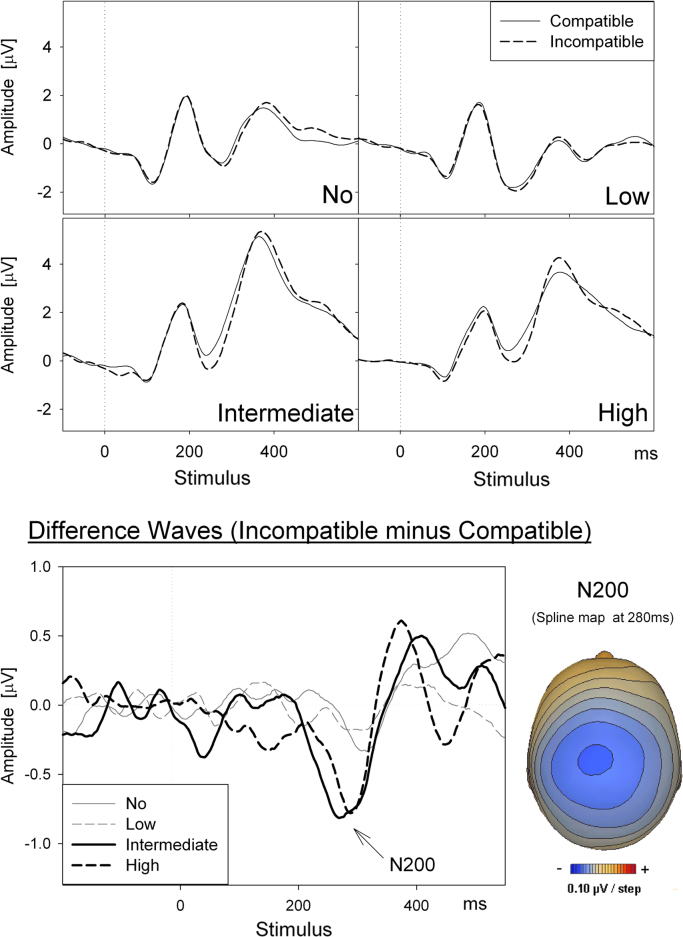
Stimulus-locked ERP waveforms depicting the N200 component (averaged across FCz and Cz electrodes). Top panels: compatible and incompatible trials superimposed in each graph for each of the four musical practice levels (no, low, intermediate, and high). Bottom panels: Stimulus-locked difference waves (incompatible minus compatible trials) with the four musical practice levels superimposed (bottom left), and the corresponding topographic voltage map for the N200 component, using the difference waveforms averaged across musical practice level (bottom right).

The N200 amplitude was larger in the Simon (−0.80 μV) than in the Stroop task (−0.08 μV), *F*(1,31)=10.60, *p*=.003, *η*_*p*_^2^=.26. More important for present purpose, the N200 amplitude increased (became more negative) with increasing levels of musical skills (see Fig. 3), *F*(linear: 1,31)=5.05, *p*=.032.

#### 3.2.2 ERN

**Fig. 4:**
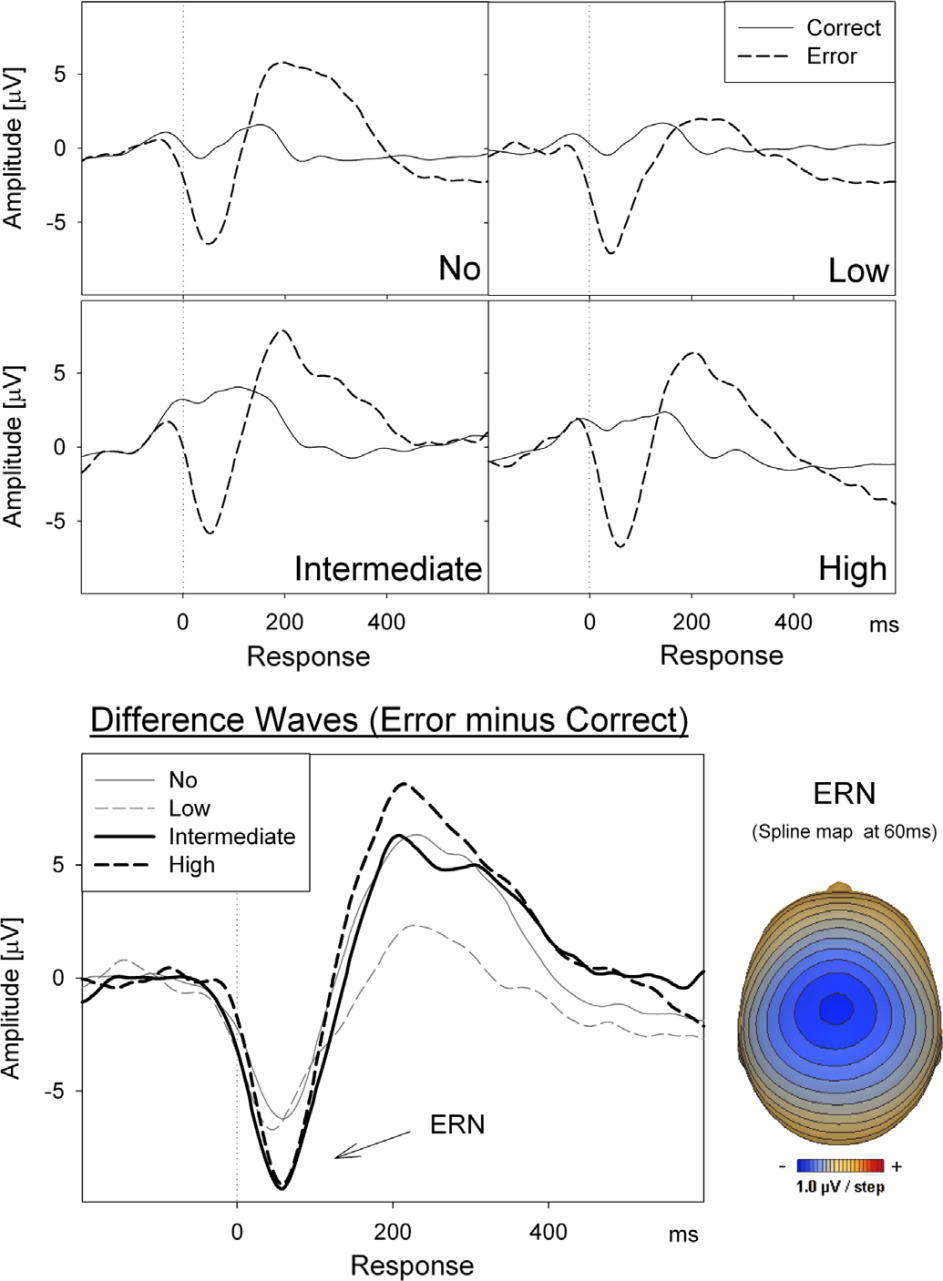
Response-locked ERP waveforms depicting the ERN component, (averaged across FCz and Cz electrodes). Top panels: correct and error trials superimposed in each graph for each of the four musical practice levels (no, low, intermediate, and high). Bottom panels: response-locked difference waves (error minus correct trials) with the four musical practice levels superimposed (bottom left), and the corresponding topographic voltage map for the ERN component using the difference waveforms averaged across musical practice level (bottom right).

The amplitude of the ERN increased (became more negative) with increasing levels of musical skill, *F*(linear: 1,31)=4.67, *p*=.039, see Fig. 4.

## 4 Discussion

The aim of the present study was to investigate the effects of musical training on behavioural and neural mechanisms involved in conflict and post-error adjustment. The amount of musical practice was positively associated with response speed. This result suggest that higher levels of musical training might result in more efficient information processing in general (indicated by faster overall speed across tasks without accuracy tradeoff), and confirms earlier reports indicating a positive link between mental speed and musical ability (e.g., Gruhn et al., 2003). More important for present purposes, higher levels of musical practice were also associated with a better engagement of cognitive control processes, as indicated by more efficient error and conflict detection (larger ERN and N2 for musicians than non-musicians), and reduced post-error interference and post-conflict processing adjustments. Importantly, these results cannot be explained by an overall difference in response speed between practice groups. More specifically, we found similar effects in both reaction times and error rates, with error rates overall to not differ between groups. Also, although overall response speed increased with increasing levels of musical practice across tasks, group differences in posterror slowing were only observed for the Stroop task but not the Simon task. The present results suggest that high levels of musical training are associated with a better ability to detect errors and conflicts and a reduced reactiveness to these detected problems. This might, at first sight, seem counterintuitive. Should one not predict larger control adjustments, when conflict and error evaluation is more effective? However, let's imagine an expert music performer. In a highly evaluative concert situation, it is very important to constantly monitor one's own performance, but not to be overtly affected by mistakes as to minimize the chance for the listeners to recognize such mistakes. A number of researchers previously investigated corrective behaviors in piano players. For example, Palmer and Drake (1997) found reduced corrective responses after performance errors in advanced adult players compared to beginner and intermediate child players. In other words, when a mistake occurred during performance, inexperienced players would stop shortly after the mistake and repeat the section where the mistake happened, whereas advanced players would rarely correct their mistakes but continue to play. However, one might argue that a global performance strategy (stop after the error and repeat a section) might be not directly comparable to local error detection and adjustment processes in an ongoing task. Maidhof, Vavatzanidis, Prinz, Rieger, and Koelsch (2010) and Loehr, Kourtis, Vesper, Sebanz and Knoblich (2013) investigated local performance monitoring processes in pianists during active piano playing tasks. Neither study reported response slowing after altered auditory outcomes. Importantly and in line with present findings, both studies found significant brain correlates indicating the detection of altered outcomes, suggesting that the lack of posterror slowing was not due to an inability to monitor ongoing performance. However, from these findings one should of course not infer that pianists’ overt behaviour is not affected by errors. Several researchers have suggested that instrumental musicians might be able to detect own errors before the error execution (Ruiz et al., 2009, Maidhof et al., 2009, Palmer et al., 2012). More specifically, these studies reported pre-error and error slowing as well as less forceful error responses during piano performance in expert pianists. In addition, Maidhof et al., and Ruiz et al. found evidence for a pre- ERN, indicating the detection of errors about 70 ms before overt error commission. Combined, these findings suggest that musicians might be able to respond extremely fast to errors, which enables them to minimize the effects of errors on posterror performance and generally adjust their behaviour more effectively in conflict-rich contexts.

If active engagement in musical activity, including extensive instrumental practice, can potentially increase frontal brain mediated error and conflict monitoring functions, such activity could be used as a powerful intervention that may remediate or slow age related cognitive decline in frontal brain functions (e.g., Sluming et al., 2002). Previous research showed reduced error monitoring related ACC activity in healthy middle-aged and older participants (Band and Kok, 2000, Falkenstein et al., 2001, Gehring and Knight, 2000, Mathalon et al., 2003, Mathewson et al., 2005, Nieuwenhuis et al., 2002, Strozyk and Jentzsch, 2012) and it has been suggested that these activity reductions might reduce older adults ability to evaluate and respond to errors (for a review, see Nieuwenhuis et al., 2002).

However, it is important to be cautious in interpreting our findings in support of a causal link between musical activity and effectiveness of frontal brain functions, as unspecific group selection confounds might explain possible differences between participant groups. Some researchers argue that, although links between music training and cognitive functioning are relatively well established, the direction of causation remains unclear (e.g., Schellenberg, 2004, Schellenberg, 2011). As Schellenberg (2011) pointed out, high functioning individuals that will perform generally well on many cognitive tasks, might also be more likely to learn an instrument in addition to participating in other activities. The author suggests that links between musical activity and cognitive functioning are best explained by innate differences with only weak contributions of learning and experience. However, others argue that musical activity does have the potential to actually *cause* differences in brain activity and cognitive functioning (e.g., Pascual-Leone, 2001, Schlaug, 2001, Wan and Schlaug, 2010). Our study cannot adequately contribute to this debate, as we used a simple cross-sectional design. Nevertheless, in an attempt to reduce potential sample biases in our study we chose a very selective participant sample: all participants were drawn from our university population (students or employees) with a comparable educational background and no significant differences in general mood. This of course strongly restricts the generalizability of our findings, but we feel that the benefits of investigating group differences within a highly homogeneous population outweight potential costs. In addition, there is no obvious reason to propose that the cognitive mechanisms we investigated in this study should be specific to our particular sample.

The present results have also theoretical implications for models of error/conflict detection. Traditional models of conflict/error detection assume a direct link between error detection and subsequent control adjustments, more specifically, that increased error/conflict signals result in stronger posterror and post-conflict adjustments (e.g., Gehring et al., 1993, Debener et al., 2005, West and Travers, 2008). Several recent publications have questioned such a direct link, proposing that control adjustments, although informed by error/conflict detection processes, rely on other factors such as external context (e.g., Larson & Clayson, 2011; Picton, Saunders, & Jentzsch, 2012). Here we provide further evidence for this idea, suggesting that an increased ability to detect of errors and conflicts does not necessarily trigger larger control adjustments (see also Loehr et al., 2013; Maidhof et al., 2009), but in contrast, can make behavioral responses after experience of conflicts and errors more efficient and less reactive.

An important aspect of this study is the choice of amateur rather than professional musicians. Most previous studies reporting cognitive and structural brain changes related to musical activity used professional musicians with extensive levels of accumulated practice time. Here we show that already moderate levels of musical activity are associated with improved executive functioning when performing basic non-musical cognitive task. This is particularly important if musical activity were to be suggested as a realistic intervention method to slow or even prevent age-related decline in frontal brain functioning. Furthermore, people who have never played an instrument or felt too old to start engaging in such an activity might be encouraged to take up music, and the associated benefits for physical and mental functioning could be much more far-reaching than proposed in this study.

## Figures and Tables

**Table:** 

Music experience questionnaire	Participant ID: ……

Age: …………..	

Sex: ……………	

Handedness (left or right): …………	

Years of Education: ……………...	

**Table:** 

Instrument	Number of years played	Accumulated Practice time	Start age

			

			

			

**Table:** 

Instrument	Number of years played	Accumulated Practice time	Start age	End age

				

				

				

**Table:** 

Instrument	Grade level	Age at which that grade was achieved

		

		

		

**Table:** 

Knowledge of music history:	1	2	3	4	5

Knowledge of music theory:	1	2	3	4	5

Ability to read music:	1	2	3	4	5

Overall music ability:	1	2	3	4	5

**Table:** 

Any other comments to music-related activities: ……………………………………………………………



How many hours per week to you on average listen to music?

…………………………………………………………………



List any other non-music-related hobbies you have: …………………………….

………………………………………………………………………



How many hours per month do you roughly spend on each of these

1. ………….. 2…………….. 3. ………….. 4…………….. 5……

## Appendix

Do you **currently** play or learning to play a musical instrument? YES/No

If yes,

Have you ever, **in the past** played or learned to play an instrument **but have stopped** playing it? YES/No

If yes,

Did you complete formal exams in the above instruments/voice: YES/No

If yes,

On a scale of 1 to 5, rate the following

(1=None or Not Able; 2=Limited; 3=Average; 4=Above Average; 5=Extensive or Very Able).

## Footnotes

1 In order to account for the differences in overall speed between groups, RT data were normalized (condition mean divided by overall mean for a given participant). The interaction between musical practice level, previous compatibility and current compatibility remained significant in the normalized RT data, F(1, 32) =2.99, *p*=.046.
